# Favipiravir versus other antiviral or standard of care for COVID-19 treatment: a rapid systematic review and meta-analysis

**DOI:** 10.1186/s12985-020-01412-z

**Published:** 2020-09-24

**Authors:** Dhan Bahadur Shrestha, Pravash Budhathoki, Sitaram Khadka, Prajwol Bikram Shah, Nisheem Pokharel, Prama Rashmi

**Affiliations:** 1 Department of Emergency Medicine, Mangalbare Hospital, Morang, Nepal; 2 Dr Iwamura Memorial Hospital, Bhaktapur, Nepal; 3 Shree Birendra Hospital, Nepalese Army Institute of Health Sciences, Kathmandu, Nepal; 4 Nepal Medical College and Teaching Hospital, Kathmandu, Nepal; 5 KIST Medical College and Teaching Hospital, Lalitpur, Kathmandu, Nepal

**Keywords:** Antiviral agents, COVID-19, COVID-19 drug treatment, Favipiravir, Severe acute respiratory syndrome coronavirus-2

## Abstract

**Background:**

The COVID-19 causing coronavirus is an enveloped RNA virus that utilizes an enzyme RNA dependent RNA polymerase for its replication. Favipiravir (FVP) triphosphate, a purine nucleoside analog, inhibits that enzyme. We have conducted this systematic review and meta-analysis on efficacy and safety of the drug FVP as a treatment for COVID-19.

**Methods:**

Databases like Pubmed, Pubmed Central, Scopus, Embase, Google Scholar, preprint sites, and clinicaltirals.gov were searched. The studies with the standard of care (SOC) and FVP as a treatment drug were considered as the treatment group and the SOC with other antivirals and supportive care as the control group. Quantitative synthesis was done using RevMan 5.4. Clinical improvement, negative conversion of reverse transcription-polymerase chain reaction (RT-PCR), adverse effects, and oxygen requirements were studied.

**Results:**

We identified a total of 1798 studies after searching the electronic databases. Nine in the qualitative studies and four studies in the quantitative synthesis met the criteria. There was a significant clinical improvement in the FVP group on the 14th day compared to the control group (RR 1.29, 1.08–1.54). Clinical deterioration rates were less likely in the FVP group though statistically not significant (OR 0.59, 95% CI 0.30–1.14) at the endpoint of study (7–15 days). The meta-analysis showed no significant differences between the two groups on viral clearance (day 14: RR 1.06, 95% CI 0.84–1.33), non-invasive ventilation or oxygen requirement (OR 0.76, 95% CI 0.42–1.39), and adverse effects (OR 0.69, 0.13–3.57). There are 31 randomized controlled trials (RCTs) registered in different parts of the world focusing FVP for COVID-19 treatment.

**Conclusion:**

There is a significant clinical and radiological improvement following treatment with FVP in comparison to the standard of care with no significant differences on viral clearance, oxygen support requirement and side effect profiles.

## Background

The outbreak of a novel coronavirus named severe acute respiratory syndrome coronavirus-2 (SARS-CoV-2) started in Wuhan, China, in late December 2019. The COVID-19 caused by such a virus was declared a global pandemic by WHO on 11th of March 2020 [1]. The number of cases and mortality that the virus has claimed around the globe is astronomical. As of 26 August 2020, the number of confirmed cases and deaths reported has reached 23,752,965 and 815,038 respectively [2]. This virus is getting transmitted mainly via respiratory tracts through droplets or respiratory secretions. The disease is characterized by asymptomatic to flu-like mild respiratory symptoms including shortness of breath (SOB) leading to pneumonia, acute respiratory distress syndrome (ARDS), and even multiple organ dysfunction in severe cases [3]. The coronavirus is an enveloped, non-segmented positive-sense RNA virus that utilizes an enzyme RNA dependent RNA polymerase (RdRp) for its replication which could be a potential target for the treatment development [4].

The road to discovering the effective prophylaxis and treatment is still an ongoing process. Numerous trials of medications of different categories have been conducted but none have succeeded to show promising results for effective treatment [5, 6]. Some of the repurposed drugs like remdesivir are being utilized along with supportive care for the management of COVID-19 in different clinical settings.

Favipiravir (FVP) triphosphate, a purine nucleoside analog, competitively inhibits the enzyme RdRp. It has shown activity against influenza viruses, RNA viruses associated with viral hemorrhagic fever, and even against SARS-CoV-2 in vitro [7]. The evidence regarding FVP is relatively low as there have only been a handful of studies regarding its efficacy and safety among COVID-19 patients. We conducted this systematic review and meta-analysis to evaluate the efficacy and safety of the drug FVP as a treatment for COVID-19.

## Objective

To determine the clinical improvement following the treatment with FVP in the cases of COVID-19, duration to attaining and percentage that attained negative conversion of RT-PCR following the treatment, adverse effects that were seen during the treatment, oxygen and mechanical ventilation requirements following the treatment.

## Methods

We used PRISMA for the systematic review of available literature [8].

### Criteria for considering studies for this review

#### Types of studies

We included studies that were done to determine the safety and efficacy of FVP along with the standard of care (SOC) for COVID-19 diagnosed cases based on guidelines in comparison to the control group receiving standard of care alone. We only included the case series with more than 5 patients, randomized controlled trials, controlled clinical trials, prospective and retrospective studies where FVP was used in the management of COVID-19 patients in the qualitative analysis. Only the studies with both the treatment and the control groups were included in quantitative synthesis.

#### Types of participants

The studies had patients with COVID-19 diagnosed as per guidelines who were enrolled either in FVP and SOC compared to standard of care alone in quantitative analysis.

#### Types of interventions

FVP along with the SOC was taken in the treatment arm and SOC alone in the control arm. SOC included other antivirals, respiratory support, antibiotics, immunomodulators, and herbal medicines.

#### Types of outcome measures

Our outcomes of interest were clinical improvements following the treatment with FVP in cases of COVID-19; negative seroconversion of RT-PCR; adverse effects that were seen during the treatment; oxygen and mechanical ventilation requirements.

#### Outcomes

The parameters for clinical improvements were symptomatic and radiological improvements (in CT scan), and clinical deterioration at 7 and 14 days after treatment between the treatment and control group. We also compared overall adverse effects that had occurred during the treatment and respiratory support requirements between the treatment and control groups. We also compared the time to negative RT-PCR and the percentage of negative RT-PCR at day 7 and 14 following treatment.

### Search methods for identification of studies

Studies were independently screened by two reviewers (DBS and PB) using COVIDENCE and data were extracted for both quantitative and qualitative synthesis. The conflicts were resolved by taking the opinion of the third reviewer (NP). Assessment of biases and cross-checking of the selected studies were done by another reviewer (SK).

#### Electronic searches

We have included the electronic search strategy in Additional file 1.

### Data collection and analysis

Databases like Pubmed, Pubmed central, Scopus, Embase, Google Scholar, bioRxiv, medRxiv, and clinicaltirals.gov were searched until 20th August, 2020. We decided to include the preprints because the studies on FVP are actively ongoing with very few papers published in academic journals. We extracted data for quantitative synthesis and analyzed it using RevMan 5.4.

#### Selection of studies

We included RCTs, controlled clinical trials, prospective and retrospective observational studies for all case series with more than 5 patients for our qualitative analysis in which FVP was used in the treatment of COVID-19 patients with sufficient details on outcomes. We included studies with the treatment groups in which patients received FVP and SOC in the treatment group and SOC alone in the control group for quantitative analysis. Studies lacking control groups were excluded in the quantitative analysis. We excluded studies where the outcomes of the patients receiving favipiravir were not properly defined. Case reports, reviews, protocols, in-vitro studies, and letters to editors were also excluded.

#### Data extraction and management

We evaluated the quality of the studies and included the outcome of interest in the quantitative synthesis.

#### Assessment of risk of bias in included studies

**Fig. 1:**
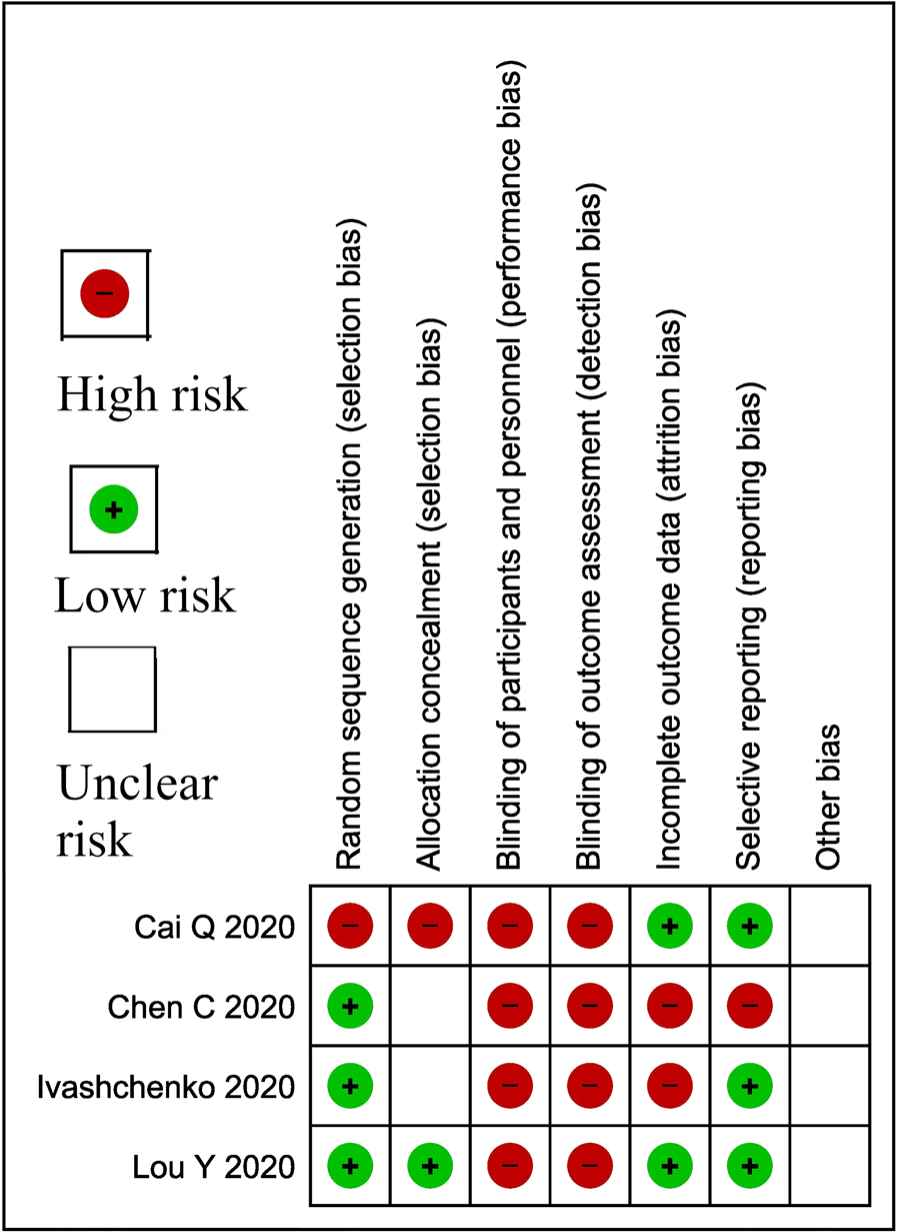
Risk of bias assessment of trials

We used the Cochrane risk of bias (ROB) tool to analyze the risk of bias shown in Fig. 1 [9]. We used the NHLBI (National Heart, Lung, and Blood Institute) quality assessment tools (Additional file 2) to assess the risk of bias in observational studies and case series (Table 1) [10]. We used the RevMan 5.4 for the creation of risk-of-bias plots.

**Table 1:** NHLBI assessment of observational studies and case series

Study	Study type	Score	Percentage	Quality
Çalik BaŞaran et al. [11]	Prospective observational study	10/14	71.4	Good
Doi et al. [12]	Case series	6/9	66.66	Good
Irie et al. [13]	Case series	6/9	66.66	Good
Rattanaumpawan et al. [14]	Retrospective observational study	8/14	57.1	Fair
Yamamura et al. [15]	Prospective single center study	10/14	71.4	Good

Good if they fulfilled 60–100% of the tool items, fair if 50–59% or Poor if 0–49%

#### Assessment of heterogeneity

We assessed the heterogeneity using the I-squared (I^2^) test. We used the Cochrane Handbook for Systematic Reviews of Interventions for interpretation of I^2^ test done as follows based on “0–40%: might not be important; 30% to 60% may represent moderate heterogeneity; 50% to 90%: may represent substantial heterogeneity; 75% to 100%: considerable heterogeneity [16]. The importance of the observed value of I^2^ depends on (1) magnitude and direction of effects and (2) strength of evidence for heterogeneity (e.g. *P* value from the chi-squared test, or a confidence interval for I^2^).”

#### Assessment of reporting biases

We assessed the reporting biases through predetermined outcome reporting documentation.

#### Data synthesis

We did a statistical analysis using RevMan 5.4 software. We used Risk Ratio (RR)/ Odds Ratio (OR) for outcome estimation whenever appropriate with 95% Confidence Interval (CI). We used the fixed/random-effects model as per the heterogeneities. We assessed the heterogeneity using the I^2^ test. We analyzed the mean differences among the two groups for the duration of viral clearance using the median, sample size, and interquartile range whenever the means and standard deviations were not provided in the study [17].

#### Subgroup analysis and investigation of heterogeneity

In the case of heterogeneity, we tried the inverse variance, random-effect model. We then ran an analysis excluding non-randomized study to evaluate their impact on the overall result wherever appropriate. We presented Forest plots to visualize the degree of variation between studies.

#### Sensitivity analysis

For sensitivity analysis, we examined the effect of study based on their type (RCT and non-RCT) by excluding non-RCT studies when appropriate and re-running the analysis to find any differences.

## Results

### Qualitative synthesis

**Fig. 2:**
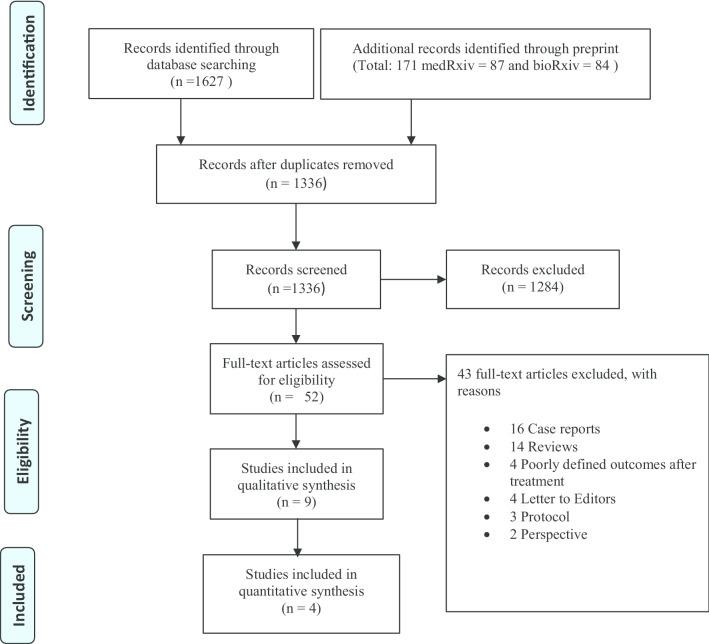
PRISMA flow chart

We identified a total of 1798 studies after searching the electronic databases. After the removal of 462 duplicates, the title and abstracts of 1336 studies were screened. We excluded 1284 studies after title and abstracts screening and 52 articles were assessed for full-text eligibility. A total of 43 articles were excluded for definite reasons. We included 9 studies in our qualitative study (Fig. 2). The summary of studies is discussed in Table 2.

**Table 2:** Qualitative synthesis of selected studies

Study, Year	Population	Intervention	Comparator	Outcome
Cai et al. [18] 2020, Open label controlled study, China	Total: 80 T: 35 C: 45 Sex: F = 45, M = men (35 of 80) History: Median age (IQR) 47 (35.75–61) *Inclusion criteria* Aged 16–75 years old; nasopharyngeal swabs samples tested positive for the novel coronavirus RNA Duration from disease onset to enrolment was less than 7 d Willing to take contraception during the study and within 7 d after treatment No difficulty in swallowing the pills *Exclusion* Severe clinical condition (meeting one of the following criteria) Resting respiratory rate greater than 30 per minute Oxygen saturation below 93%, oxygenation index < 300 mm Hg Respiratory failure, shock, and/or combined failure of other organs that required ICU monitoring and treatment) Chronic liver and kidney disease and reaching end stages Previous history of allergic reactions to FPV or LPV/RTV Pregnant or lactating women Women of childbearing age with a positive pregnancy test, breastfeeding, miscarriage, or within 2 weeks after delivery; Participated in another clinical trial against SARS-CoV-2 treatment currently or in the past 28 d	*Treatment group* FPV was 1600 mg twice daily on Day 1 and 600 mg twice daily on days 2–14 Medications were given till viral clearance was confirmed or 14 days had passed Patients received IFN-a1b 60 mg twice daily by aerosol inhalation	*Control group* LPV/RTV was LPV 400 mg/RTV 100 mg twice daily Medications were given till viral clearance was confirmed or 14 days had passed Patients received IFN-a1b 60 mg twice daily by aerosol inhalation	Median time of viral clearance T: 4 d (IQR: 2.5–9); C: 11 d (IQR:8–13) D8: RT-PCR negative for viral clearance T:26/35; C:17/45 D16: RT-PCR negative for viral clearance T:33/35; C:33/45 CT improvement D4: T:8/35; C:8/45 D9: T:18/35; C:16/45 D14 T:32/35; C:28/45 CT worse D14: T:1/35; C:9/45 Total number of adverse reactions T:4/35; C:25/45
Calik Basaran et al. [11] 2020, Prospective observational study, Turkey	Total: 174 M: 91, F: 83 Mild: 35 Moderate: 107 Severe: 32 *Inclusion criteria* Adult patients (More than or equal to 18 years) hospitalized in COVID ward from March 20 to April 30, 2020 *Exclusion criteria* Critically ill patients with sepsis or ARDS requiring ICU at the time of admission	32 patients received favipiravir, two patients received favipiravir monotherapy while 30 received it to the initial regimen or with other antivirals 23 patients received HCQ alone while 113 received HCQ + AZT in addition to other supportive treatment		Median time to defervescence days HCQ: 1 (0–4); HCQ + AZT: 1 (0–11); FVP: 3 (0–8) Median time to clinical improvement on therapy HCQ: 1 (1–6); HCQ + AZT: 1.5 (1–11); FVP: 6 (1–10) Median duration LOS HCQ: 2 (1–21); HCQ + AZT: 4 (1–15); FVP: 7.5 (2–24) *Nausea/vomiting* HCQ: 1; HCQ + AZT: 5; FVP: 5 *Elevation of transaminase* HCQ: 1; HCQ + AZT: 3; FVP: 10
Chen et al. [19] 2020, RCT, China	Total: 236 T: 116 C: 120 *Inclusion* Age 18 years or older Voluntarily provided informed consent Initial symptoms were within 12 days Diagnosed as COVID-19 pneumonia *Exclusion* Allergic to FVP or Arbidol Increased ALT/AST (> 6 × upper limit of normal range) or with chronic liver disease (cirrhosis at grade Child–Pugh C) Severe/critical patients whose expected survival time were < 48 h Pregnant female HIV infected Considered unsuitable by researchers for patient’s interest	*Treatment group* Patients received FVP (1600 mg, twice the first day followed by 600 mg, twice daily, for the following days plus standard care for 7 days	*Control group* Patients received Arbidol (200 mg, three times daily) plus standard of care for 7 days Standard of care included traditional Chinese herbal medicine, antibiotics, additional antiviral treatment, immunomodulatory drugs, steroids, psychotic drugs, nutrition support, cardiovascular drugs, supportive oxygen, noninvasive positive pressure ventilation (NPPV) or invasive ventilation	D7 Clinical Recovery T: 71/116; C: 62/120 Clinical deterioration (new dyspnea) T: 13/116; C: 15/120 D7 NIMV OR Oxygen support T: 21/116; C:27/120 Total number of adverse reactions T:37/116; C:28/120 Respiratory failure T: 1/116; C: 4/120 No mortalities
Doi et al. [12] 2020, Case series, Japan	Total: 11 M: 10 F: 1 Comorbidities HTN 4, DM 3, COPD 1 and Cancer 1 Age: 60–69 All patients admitted to ICU 8 patients required MV and 3 required VV-ECMO	Treatment with nafamostat mesylate [0.2 mg per kg per hour by continuous intravenous infusion, median treatment 14 days (IQR, 10 to 14 days)] and FVP [3600 mg on day 1 and at 1600 mg per day on day 2 and subsequently median treatment 14 days (IQR, 12 to 14 days)		Mortality: 1; 7 Patients weaned from MV Discharge from ICU: 9 Discharge from hospital: 7 Adverse effect: 1 (Hyperkalemia)
Lou et al. [20] 2020, Open-label RCT, China	Total: 29 T = 9 and C = 10 T = FPV and C = Control Sex: F = 5, M = 14 History: Median age (SD) T = 58.0 (8.1); C = 46.6 (14.1) Inclusion: All RT-PCR diagnosed Exclusion: Patients who dint complete the dosage of the medication Previous history of malignancy, COPD, renal insufficiency and hepatic insufficiency	*Treatment group* Baloxavir marboxil or FVP to the current standard antiviral treatment was randomly allocated (1:1:1) *FVP group* FVP was used in combination with the existing antiviral treatment. The first dose was 1600 mg or 2200 mg orally, followed by 600 mg each time, three times a day, and the duration of administration was not more than 14 days *Baloxavir group* The dose was 80 mg OD on Day 1 and 4 and if patients are positive it can be given on Day 7 but no more than 3 doses should be given Both groups received existing antiviral treatment including lopinavir/ritonavir (400 mg/100 mg, twice a day orally) or 8 darunavir/cobicistat (800 mg/150 mg, four times a day orally) and arbidol (200 mg, thrice a day orally) along with interferon-alpha inhalation	*Control group* Patients received existing antiviral treatment including lopinavir/ritonavir (400 mg/100 mg, twice a day orally) or 8 darunavir/cobicistat (800 mg/150 mg, four times a day orally) and arbidol (200 mg, thrice a day orally) along with interferon-alpha inhalation	Viral negative in Day 7 T(FVP group): 4/9; C: 5/10 Viral negative in Day 14 T(FVP group): 7/9; C: 10/10 Clinical improvement Day 14 T(FVP group): 5/9; C: 5/10 Day 7 T(FVP group): 2/9; C: 1/10 D14 Discharge T(FVP group): 4/9; C: 4/10 Time to clinical improvement—median days (IQR) T(FVP group): 14 (6–38); C: 15 (6–24) Time to viral negative-median days (IQR) T(FVP group): 9 (2–34; C: 9 (1–13) D14 NMV OR Oxygen support T: 3/9; C: 4/10
Ivaschenko et al. [21] 2020, Multi center, open label randomized Phase II/ III controlled trial, Russia	Total: 60 Randomization in 1:1:1 in three groups comparable in demographic and baseline characteristics Intention to treat analysis was done *Inclusion criteria* Hospitalized men and non-pregnant women of 18 years or older who signed the informed consent form, had moderate PCR-confirmed COVID-19 and were able to administrate the drug orally and willing to use adequate contraception during the study and 3 months after its completion	*Treatment group* One group received either AVIFAVIR 1600 mg BID on Day 1 followed by 600 mg BID on Days 2–14 (1600/600 mg) Other group received AVIFAVIR 1800 mg BID on Day 1 followed by 800 mg BID on Days 2–14 (1800/800 mg) Patients receiving AVIFAVIR did not receive other antivirals or antimalarial drugs	*Control group* Control group received standard of care according to national guideline 15 patients reveived HCQ or CQ 1 patient received Lopinavir and ritonavir 4 patients did not receive etiotropic treatment	Viral clearance Day 5 TG(FVP group): 25/40; CG: 6/20 Day 10 TG: 37/40; CG: 16/20 Median time to body temperature normalization TG: 2 days (IQR 1–3); CG: 4 days (IQR 1–8) CT improvement at day 15 TG: 36/40; CG: 16/20 Adverse effects TG: 15/40; CG: 5/20 Common side effects were diarrhea, nausea, vomiting, chest pain and increase in liver transaminase levels *Early drug discontinuation in 2 patients out of 40 in treatment group* Mortality: 2 in TG Discharge AVIFAVIR 1600/600: 13/20 AVIFAVIR 1800/800: 17/20 CG: 17/20
Irie et al. [13] 2020, Case Series, Japan	Total: 7 M: 5 F: 2 Comorbidities HTN: 3 DM: 2 Hyperuricemia: 2 Others included BPH, gout, and fibroid Inclusion: Critically ill patients admitted to ICU under mechanical ventilation	Patients were given 1600 mg FPV on day 1 and 600 mg from day 2–5		Clinical improvement: 3/7 At Day 7: 1/7 No requirement for mechanical ventilation: 1/7 At Day 14: 3/7 Weaned from mechanical ventilation: 3/7 No oxygenation support: 2/7 Adverse effect: 1/7 (Increase in transaminase)
Rattanaumpawan et al. [14] 2020, Observational study, Thailand	Total: 247 T: 63 C: 184 Inclusion: Patients aged at least 18 years who had RT-PCR-confirmed SARS-CoV-2 based on a respiratory specimen (nasopharyngeal, oropharyngeal, sputum, endotracheal aspirate, or bronchoalveolar lavage sample) and received at least one dose of FVP Exclusion: Patients who expired or were discharged within 24 h of hospital stay	*Treatment group* Patients received the median loading dose of FVP of 47.4 (29.1–71.1) MKD along with the standard of care, and one-third of 176 enrolled patients (33.3%) received a loading dose of ≤ 45 MKD The median maintenance 177 dose of FVP was 17.9 (10.9–26.7) MKD, and 76.2% of the subjects received a 178 maintenance dose of ≤ 15 MKD The median duration of FVP therapy was 12 (2–17) days Standard of care includes protease inhibitors, hydroxychloroquine, azithromycin, steroid, respiratory support, and tocilizumab *Control group* Patients received standard of care including protease inhibitors, hydroxychloroquine, azithromycin, steroid, tocilizumab, and respiratory support		Outcomes of treatment groups have been only reported. N = 63 Clinical improvement D7: 42/63 No requirement of oxygen supplementation: 25/63 D14: 54/63 No requirement of oxygen supplementation: 27/63 D28: 57/63 No requirement of oxygen supplementation: 27/63 Mortality D14: 1 D28: 3 Adverse drug reaction 39/63 Most common diarrhea (34) and hepatitis (4)
Yammamura et al. [15] 2020, Prospective single center study, Japan	Total: 13 M: 9 F: 4 Mean age: 63 All patients were mechanically ventilated at the time of admission Comorbidities HTN 8, DM 7, Bronchial asthma 1, sleep apnea syndrome 3	FPV (3600 mg on day 1, 1600 mg from day 2 to day 14), methylprednisolone (1000 mg for 3 days), and low molecular weight (2000 IU every 12 h) or unfractionated heparin (10,000–12,000 IU/day). Methylprednisolone administration was begun on the 5th day from initial FPV administration. Heparin and dexmedetomidine were administered after intubation and mechanical ventilation		Survival: 12 Mortality: 1 Improvement in IL-6 5 days after FPV therapy, PaO2/Fi02 in a week after FVP therapy

*ALT* alanine transaminase, *AST* aspartate transaminase, *BID* twice a day, *C* control, *COPD* chronic obstructive pulmonary disease, *CG* control group, *D* day, *DM* diabetes mellitus, *FPV* favipiravir, *F* female, *Fi02* fraction of inspired oxygen, *HIV* human immunodeficiency virus, *HTN* hypertension, *IU* international unit, *ICU* intensive care unit, *IQR* interquartile range, *M* male, *MKD* mean dose per kg, *MV* mechanical ventilation, *N* total number of patients, *LPV* Lopinavir, *Pa02* partial pressure of oxygen, *RNA* ribonucleic acid, *RT-PCR* reverse transcription-polymerase chain reaction, *RTV* ritonavir, *SARS* severe acute respiratory syndrome, *T* treatment, *TG* treatment group, *VV-ECMO* veno venous extra corporeal membrane oxygenation

### Quantitative analysis

Four studies meet the criteria and are included in the quantitative synthesis. In the present meta-analysis, we have compared findings among randomized/non-randomized controlled studies to extract outcome on viral clearances, improvements or deteriorations among FVP group in comparison to COVID-19 cases getting other antivirals or SOC, duration to viral clearance, the requirement of non-invasive mechanical ventilation/ oxygen support and adverse effects.

#### FVP versus other antivirals or SOC only; effectiveness

Among the treatment groups FVP in addition to SOC versus other antivirals or SOC we have compared the duration of viral clearance (negative RT-PCR) and radiological/ clinical improvement.

##### Viral clearance

**Fig. 3:**
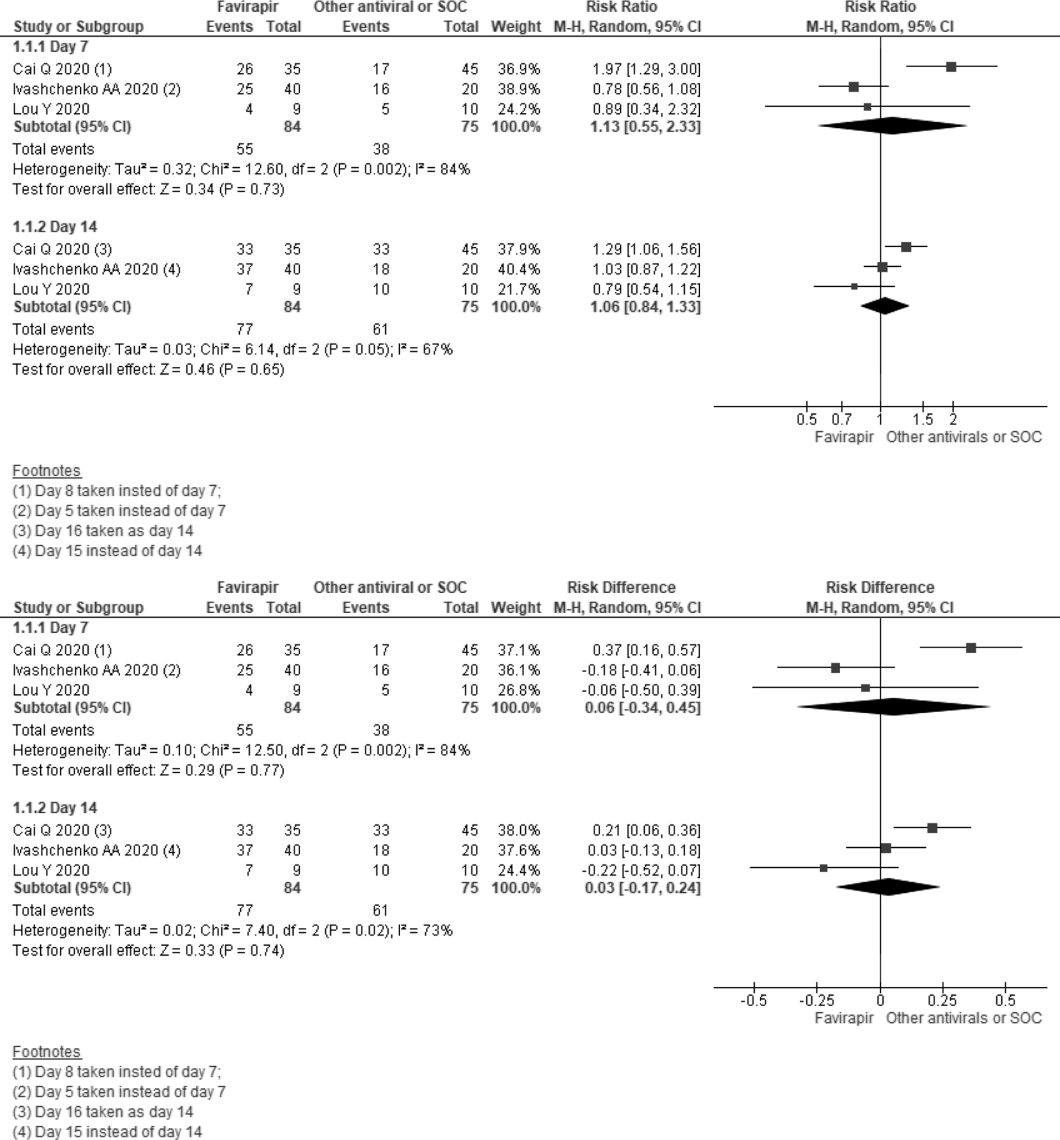
Forest plot for risk ratios and risk differences regarding FVP in addition to SOC effectiveness for viral clearance compared with other antivirals or SOC

The meta-analysis of risk ratios (RR) for FVP in addition to SOC effectiveness compared with other antivirals or SOC using random effect model among randomized and non-randomized studies showed that there were no significant differences between two groups (Day 7: RR 1.13, 95% CI 0.55 to 2.33; Day 14: RR 1.06, 95% CI 0.84 to 1.33). Also, there is no significant risk difference (RD) for viral clearance between two groups FVP in addition to SOC versus other antivirals or SOC (Day 7: RD 0.06, 95% CI − 0.34 to 0.45; Day 14: RD 0.03, 95% CI − 0.17 to 0.24) (Fig. 3). For heterogeneity, both subgroup assessments inverse variance method and excluding non-randomized study by Cai et al. [18] showed no significant changes (Additional file 3/Figs. 1 and 2).

##### Clinical/CT improvement

**Fig. 4:**
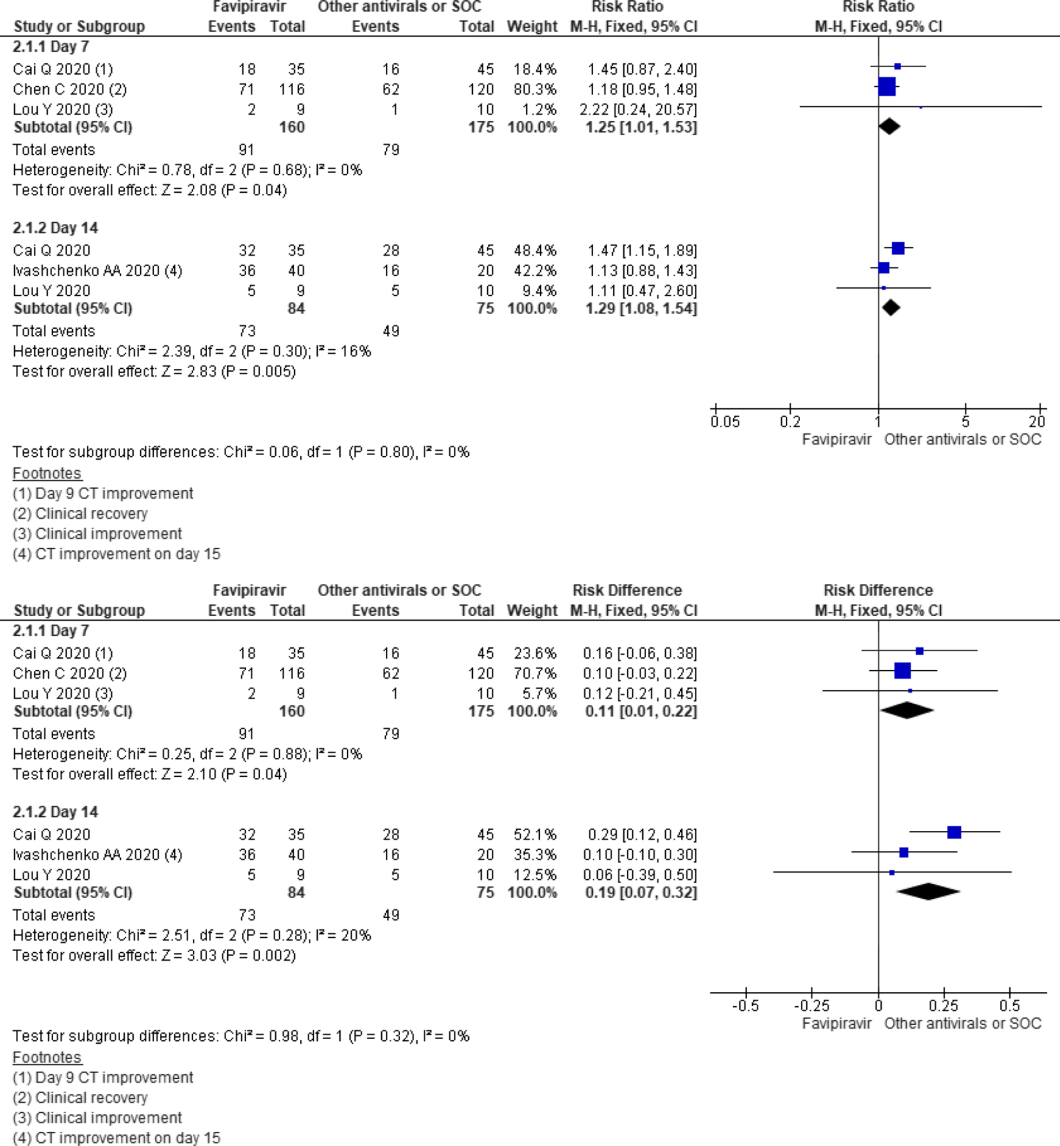
Forest plot for risk ratios and risk differences regarding FVP in addition to SOC effectiveness for clinical improvement compared with other antivirals or SOC

Among three studies, two reported clinical and two reported CT improvement, overall risk ratios (RR) for FVP in addition to SOC effectiveness compared with other antivirals or SOC alone using fixed-effect model showed that there was a significant improvement on FVP groups on both 7^th^ and 14^th^ day of treatment (Day 7: RR 1.25, 95% CI 1.01 to 1.53; Day 14: RR 1.29, 95% CI 1.08 to 1.54). Furthermore, there are similar findings on risk difference (RD) between two groups for improvement (Day 7: RD 0.11, 95% CI 0.01 to 0.22; Day 14: RD 0.19, 95% CI 0.07 to 0.32) (Fig. 4).

Clinical improvement on the 7th and 14^th^ day among randomized controlled trials after excluding non-randomized study by Cai et al. [18] showed slight improvement on favipiravir arm but statistically not significant (Additional file 3/Fig. 3).

#### FVP versus other antivirals: clinical/CT deterioration

**Fig. 5:**
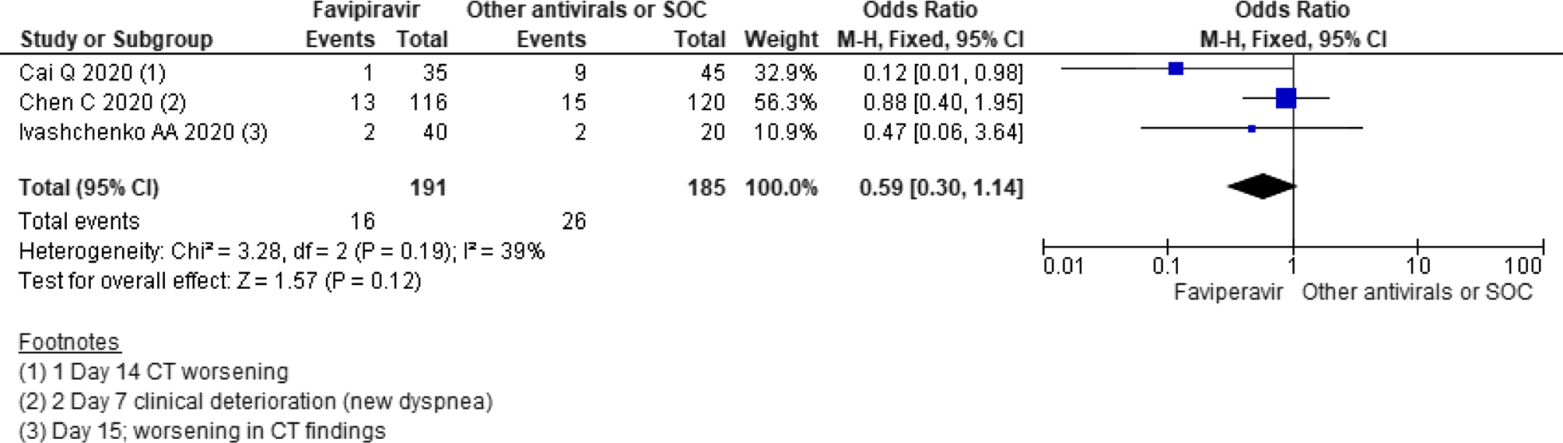
Forest plot for odds ratios regarding clinical deterioration among FVP group versus other antivirals

The meta-analysis on clinical deterioration rate at the end of study duration showed clinical deteriorations is less likely in the FVP treatment group than other antiviral agents though statistically not significant (OR 0.59, 95% CI 0.30 to 1.14; participants = 376; studies = 3; I^2^ = 39%) (Fig. 5).

#### FVP group versus other antivirals or SOC group: Oxygen support or non-invasive ventilation

**Fig. 6:**
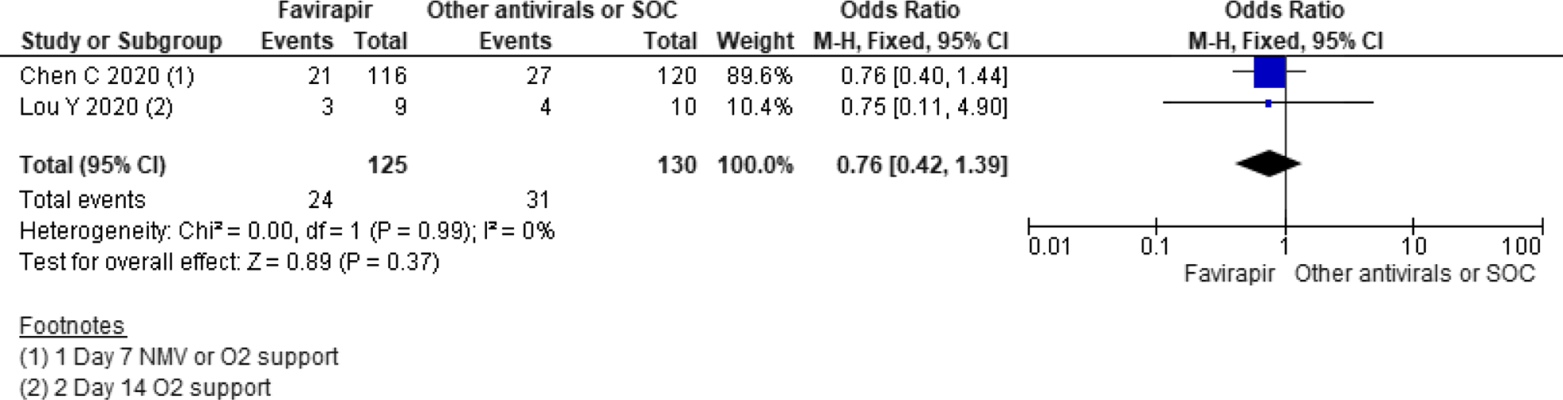
Forest plot for odds ratios requiring oxygen support or non-invasive ventilation among FVP group versus other antivirals or SOC group

Meta-analysis on the oxygen support requirements and non-invasive mechanical ventilation among included randomized studies showed decreased odds of oxygen support among FVP group but it is not statistically significant (OR 0.76, 95% CI 0.42 to 1.39; participants = 255; studies = 2; I^2^ = 0%) (Fig. 6).

#### Adverse effects

**Fig. 7:**
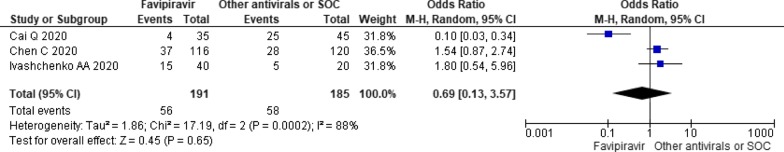
Forest plot for odds ratios for adverse effects among FVP group versus other antivirals

Meta-analysis comparing adverse effects between the treatment and the control groups showed lesser odds for adverse effect in the treatment arm but of no statistical significance (OR 0.69, 95% CI 0.13 to 3.57; participants = 376; studies = 3; I^2^ = 88%) (Fig. 7). Overall adverse effects among randomized controlled trials after excluding non-randomized study by Cai et al. [18] showed slight increase in adverse effects among favipiravir arm but statistically not significant. This may be due to heterogeneity in treatments patients might be taking other than favipiravir or other standard treatment (Additional file 3/Fig. 4).

#### Duration to convert negative RT-PCR

**Fig. 8:**
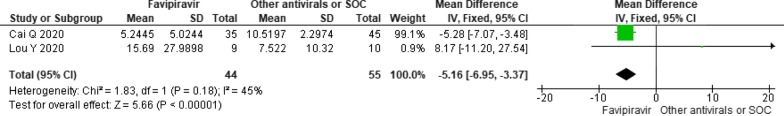
Forest plot of FVP in addition to standard of care or other anti-virals on duration for negative conversion of RT-PCR

Our meta-analysis on negative conversion of RT-PCR demonstrated approximately 5 days (MD − 5.16, 95% CI − 6.95 to − 3.37; participants = 99; studies = 2; I^2^ = 45%) earlier on treatment with FVP group (Fig. 8). Data being subject to moderate heterogeneity sensitivity assessment using the random-effect model showed no significance (MD − 2.16, 95% CI − 13.28 to 8.97). This finding, thus needs to be confirmed by further randomized studies (Additional file 3/Fig. 5).

### Clinical trials

Focusing on the safety and efficacy of FVP for COVID-19 treatment along with different parameters, there are 31 RCTs registered in different parts of the world as of 25 August 2020 (Additional file 4) [22]*.* Five of such trials have recently been completed from Egypt, Iran, and Turkey. Among the registered RCTs, 14 trials are recruiting participants, 6 trials have not yet started recruiting, and 4 trials are active but not recruiting any participants. One of the trials has been withdrawn thus not been included in this calculation. According to the location provided in 31 trials, a maximum number of trials are regulated by Turkey.

## Discussion

Our meta-analysis was focused on the assessment of the clinical outcome and adverse effects following therapy with FVP because it has emerged as one of the treatments repurposed for COVID-19. Although some promise has been shown by remdesivir and plasma therapy, the lack of highly efficacious and safe treatment for COVID-19 remains one of the biggest conundrums of the twenty-first century. Our study found that patients had a significant improvement in FVP groups on both the 7^th^ and 14^th^ day of treatment (Day 7: RR 1.25, 95% CI 1.01 to 1.53; Day 14: RR 1.29, 95% CI 1.08 to 1.54). The clinical deterioration is less likely in the FVP treatment groups than other antiviral agents (OR 0.59, 95% CI 0.30 to 1.14) following treatments though of no statistical significance. There were no significant differences between the two groups in terms of viral clearance (Day 7: RR 1.13, 95% CI 0.55 to 2.33; Day 14: RR 1.06, 95% CI 0.84 to 1.33). There were lesser odds for adverse effect in the treatment group but of no statistical significance (OR 0.69, 95% CI 0.13 to 3.57). In general, there were tolerable minor side effects like nausea, vomiting, diarrhea and an increase in transaminases and no serious life-threatening complications following the FVP treatment. The possible side effects can however not be credited to favipiravir alone because the patients in treatment groups were receiving other drugs in 3 trials except the one done by Ivashchenko et al. [21]. As this is the first meta-analysis comparing the clinical outcome and adverse effects among patients receiving FVP compared to standard of care, we could not compare our findings with other meta-analyses.

Although good promise has been shown by FVP, additional randomized double-blind clinical trials are needed to give a definite opinion about the rationale of the drug. We could only include four studies for our quantitative analysis and one of the studies among them was non-randomized. The sample size was small in our studies which could decrease the power of our study. The duration of treatment and dosages were different among various studies in qualitative analysis. Two of the RCTs that were included for our analysis had a varied duration of treatment as well. Lack of randomization may have led to selection bias in the non-randomized studies. Blinding was not applied to source studies leading to biases. Selective reporting may have been a problem in Chen’s study [19] because of the limited observation time frame. It is important to determine the appropriate dose and duration of treatment with FVP because low dose therapy is found to be a bad prognostic factor for clinical improvement and widespread variations in treatment duration among studies and lack of effective plasma concentrations of drug in critically ill patients [13, 14]. Due to the early evidence of potential benefits shown by this drug in clinical improvement as well as imaging improvement, it is necessary to conduct large-scale prospective, double-blind randomized controlled trials or wait for the result of ongoing studies to come. This will embolden the evidences led by our study and eliminate biases so that definitive advice for treatment can be given in the coming days.

## Conclusion

Our study concludes that patients had clinical and radiological improvements following the treatment with FVP in comparison to that of the standard of care though no significant differences on viral clearance, oxygen support requirement and side effect profile. The results of ongoing clinical trials should be obtained to give any definite judgment on whether the treatment with FVP is the best option among antiviral treatments for COVID-19 or not. Till then, our meta-analysis supports judicial use of FVP in clinical settings.

## Supplementary information

Additional File 1: Search strategy

Additional File 2: NHLBI Bias of observational and case series

Additional File 3: Synthesis and sensitivity assessment

Additional File 4: Clinical trials

Additional File 5: Prisma checklist

## Data Availability

The datasets analyzed during the current study are available from the corresponding author on reasonable request.

## Abbreviations

ALT: Alanine transaminase
ARDS: Acute respiratory distress syndrome
AST: Aspartate transaminase
C: Control
CI: Confidence interval
COPD: Chronic obstructive pulmonary disease
COVID-19: Coronavirus disease-19
CT: Computed Tomography
D: Day
DM: Diabetes mellitus
F: Female
FVP: Favipiravir
HIV: Human immunodeficiency virus
HTN: Hypertension
I^2^: I-squared
ICU: Intensive care unit
IQR: Interquartile range
LPV: Lopinavir
M: Male
MKD: Mean dose per kg
N: Total number of patients
NHLBI: National Heart, Lung, and Blood Institute
OR: Odds ratio
PRISMA: Preferred reporting items for systematic reviews and meta-analyses
RCTs: Randomized controlled trials
RdRp: RNA dependent RNA polymerase
ROB: Risk of bias
RR: Relative risk
RT-PCR: Reverse transcription-polymerase chain reaction
RTV: Ritonavir
SARS-CoV-2: Severe acute respiratory syndrome coronavirus-2
SOB: Shortness of breath
SOC: Standard of care
T: Treatment
