# Supplementary material for: Favipiravir versus other antiviral or standard of care for COVID-19 treatment: a rapid systematic review and meta-analysis
Source: Virol J. 2020 Sep 24;17:141. doi: 10.1186/s12985-020-01412-z (PMC7512218; doi:10.1186/s12985-020-01412-z)
Supplement: Supplementary file 1 — Search strategy [file 12985_2020_1412_MOESM1_ESM.docx]

**Pubmed**

**Number: - 113**

**("coronavirus"[mh] OR "COVID 19"[tw] OR "SARS Cov 2"[All Fields] OR "novel coronavirus"[tw]) AND (avigan or favipiravir) AND (("2006/01/01"[Date - Create]: "2020/08/20"[Date - Create])**

<https://pubmed.ncbi.nlm.nih.gov/?term=%28%28%28%28%22coronavirus%22%5BMeSH+Terms%5D+OR+%22COVID+19%22%5BText+Word%5D%29+OR+%22SARS+Cov+2%22%5BAll+Fields%5D%29+OR+%22novel+coronavirus%22%5BText+Word%5D%29+AND+%28%28%28%22favipiravir%22%5BSupplementary+Concept%5D+OR+%22favipiravir%22%5BAll+Fields%5D%29+OR+%22avigan%22%5BAll+Fields%5D%29+OR+%28%22favipiravir%22%5BSupplementary+Concept%5D+OR+%22favipiravir%22%5BAll+Fields%5D%29%29%29+AND+%28%28%222006%2F01%2F01%22%5BDate+-+Create%5D+%3A+%222020%2F08%2F20%22%5BDate+-+Create%5D%29%29&sort=>

**Pubmedcentral**

**("coronavirus"[mh] OR "COVID 19"[tw] OR "SARS Cov 2"[All Fields] OR "novel coronavirus"[tw]) AND (avigan or favipiravir)**

**Number: - 845**

<https://www.ncbi.nlm.nih.gov/pmc/?term=(%22coronavirus%22%5Bmh%5D+OR+%22COVID+19%22%5Btw%5D+OR+%22SARS+Cov+2%22%5BAll+Fields%5D+OR+%22novel+coronavirus%22%5Btw%5D)+AND+(avigan+or+favipiravir)>

**Google scholar**

**Favipiravir and covid 19 NOT HIV NOT AIDS**

**Number: - 376**

<https://scholar.google.com/scholar?hl=en&as_sdt=0%2C5&q=favipiravir+and+covid+19+NOT+HIV+NOT+AIDS&btnG=>

**Embase**

**( ( coronavirus OR covid 19 OR sars AND cov 2 OR novel AND coronavirus ) AND ( avigan OR favipiravir ) )**

**Number:- 84**

[https://urldefense.com/v3/__http://www.refworks.com/refshare2?site=029961117609200000*144431585598951518*_85,114,106,97,44,32,80,47,47,47,70,97,118,105,112,105,114,97,118,105,114,32,38,32,67,111,118,105,100,49,57,32,56,45,50,48,32,106,114,98&enc=y__;Ly8!!MuWMPV1_1eXDnA!kpQ6OZjchKR5nUJkiy4XgfQxudVeCUi_DJchr1YQBXGdABM4TmIAZ9l_n9eCYp-Q5NsgXQw$](https://urldefense.com/v3/__http:/www.refworks.com/refshare2?site=029961117609200000*144431585598951518*_85,114,106,97,44,32,80,47,47,47,70,97,118,105,112,105,114,97,118,105,114,32,38,32,67,111,118,105,100,49,57,32,56,45,50,48,32,106,114,98&enc=y__;Ly8!!MuWMPV1_1eXDnA!kpQ6OZjchKR5nUJkiy4XgfQxudVeCUi_DJchr1YQBXGdABM4TmIAZ9l_n9eCYp-Q5NsgXQw$)

**Scopus**

**( ( coronavirus OR covid 19 OR sars AND cov 2 OR novel AND coronavirus ) AND ( avigan OR favipiravir ) )**

**Number:- 209**

<https://www.scopus.com/results/results.uri?sort=plf-f&src=s&st1=+%28+coronavirus+OR+COVID+19+OR+SARS+Cov+2+OR+novel+coronavirus+%29+AND+%28avigan+OR+favipiravir%29&nlo=&nlr=&nls=&sid=d3790784c08bad79998c64ce94603ea7&sot=b&sdt=b&sl=106&s=TITLE-ABS-KEY%28+%28+coronavirus+OR+COVID+19+OR+SARS+Cov+2+OR+novel+coronavirus+%29+AND+%28avigan+OR+favipiravir%29%29&cl=t&offset=1&origin=resultslist&ss=plf-f&ws=r-f&ps=r-f&cs=r-f&cc=10&txGid=234b78f1bc374243020134cae9200990>

**medRxiv**

**Favipiravir and covid-19**

**Number:- 87**

<https://www.medrxiv.org/search/favipiravir%252Band%252Bcovid%252B19?page=1>

**bioRxiv**

**Favipiravir and covid-19**

**Number 84**

<https://www.biorxiv.org/search/covid%252B19%252Band%252Bfavipiravir%20numresults%3A75%20sort%3Arelevance-rank>

**Clinical trials.gov**

**Number 31**

<https://www.clinicaltrials.gov/ct2/results?cond=Covid19&term=favipiravir&cntry=&state=&city=&dist=>
