# Supplementary material for: Favipiravir versus other antiviral or standard of care for COVID-19 treatment: a rapid systematic review and meta-analysis
Source: Virol J. 2020 Sep 24;17:141. doi: 10.1186/s12985-020-01412-z (PMC7512218; doi:10.1186/s12985-020-01412-z)
Supplement: Supplementary file 2 — NHLBI Bias of observational and case series [file 12985_2020_1412_MOESM2_ESM.docx]

**NHLBI for observational Studies**

1. Çalik BaŞaran N et al 10/14

| **Criteria** | **Yes** | **No** | **Other (CD, NR, NA)*** |
| --- | --- | --- | --- |
| 1. Was the research question or objective in this paper clearly stated? | + |  |  |
| 2. Was the study population clearly specified and defined? | + |  |  |
| 3. Was the participation rate of eligible persons at least 50%? | + |  |  |
| 4. Were all the subjects selected or recruited from the same or similar populations (including the same time period)? Were inclusion and exclusion criteria for being in the study prespecified and applied uniformly to all participants? | + |  |  |
| 5. Was a sample size justification, power description, or variance and effect estimates provided? |  | - |  |
| 6. For the analyses in this paper, were the exposure(s) of interest measured prior to the outcome(s) being measured? | + |  |  |
| 7. Was the timeframe sufficient so that one could reasonably expect to see an association between exposure and outcome if it existed? | + |  |  |
| 8. For exposures that can vary in amount or level, did the study examine different levels of the exposure as related to the outcome (e.g., categories of exposure, or exposure measured as continuous variable)? |  | - |  |
| 9. Were the exposure measures (independent variables) clearly defined, valid, reliable, and implemented consistently across all study participants? | + |  |  |
| 10. Was the exposure(s) assessed more than once over time? | + |  |  |
| 11. Were the outcome measures (dependent variables) clearly defined, valid, reliable, and implemented consistently across all study participants? | + |  |  |
| 12. Were the outcome assessors blinded to the exposure status of participants? |  |  | NR |
| 13. Was loss to follow-up after baseline 20% or less? | + |  |  |
| 14. Were key potential confounding variables measured and adjusted statistically for their impact on the relationship between exposure(s) and outcome(s)? |  | - |  |

1. Rattanaumpawan 8/14

| **Criteria** | **Yes** | **No** | **Other (CD, NR, NA)*** |
| --- | --- | --- | --- |
| 1. Was the research question or objective in this paper clearly stated? | + |  |  |
| 2. Was the study population clearly specified and defined? | + |  |  |
| 3. Was the participation rate of eligible persons at least 50%? |  | - |  |
| 4. Were all the subjects selected or recruited from the same or similar populations (including the same time period)? Were inclusion and exclusion criteria for being in the study prespecified and applied uniformly to all participants? | + |  |  |
| 5. Was a sample size justification, power description, or variance and effect estimates provided? |  | - |  |
| 6. For the analyses in this paper, were the exposure(s) of interest measured prior to the outcome(s) being measured? | + |  |  |
| 7. Was the timeframe sufficient so that one could reasonably expect to see an association between exposure and outcome if it existed? | + |  |  |
| 8. For exposures that can vary in amount or level, did the study examine different levels of the exposure as related to the outcome (e.g., categories of exposure, or exposure measured as continuous variable)? | + |  |  |
| 9. Were the exposure measures (independent variables) clearly defined, valid, reliable, and implemented consistently across all study participants? |  | - |  |
| 10. Was the exposure(s) assessed more than once over time? | + |  |  |
| 11. Were the outcome measures (dependent variables) clearly defined, valid, reliable, and implemented consistently across all study participants? |  | - |  |
| 12. Were the outcome assessors blinded to the exposure status of participants? |  |  | NR |
| 13. Was loss to follow-up after baseline 20% or less? |  | - |  |
| 14. Were key potential confounding variables measured and adjusted statistically for their impact on the relationship between exposure(s) and outcome(s)? | + |  |  |

1. Yamamura 10/14

| **Criteria** | **Yes** | **No** | **Other (CD, NR, NA)*** |
| --- | --- | --- | --- |
| 1. Was the research question or objective in this paper clearly stated? | + |  |  |
| 2. Was the study population clearly specified and defined? | + |  |  |
| 3. Was the participation rate of eligible persons at least 50%? | + |  |  |
| 4. Were all the subjects selected or recruited from the same or similar populations (including the same time period)? Were inclusion and exclusion criteria for being in the study pre specified and applied uniformly to all participants? | + |  |  |
| 5. Was a sample size justification, power description, or variance and effect estimates provided? |  | - |  |
| 6. For the analyses in this paper, were the exposure(s) of interest measured prior to the outcome(s) being measured? | + |  |  |
| 7. Was the timeframe sufficient so that one could reasonably expect to see an association between exposure and outcome if it existed? | + |  |  |
| 8. For exposures that can vary in amount or level, did the study examine different levels of the exposure as related to the outcome (e.g., categories of exposure, or exposure measured as continuous variable)? |  | - |  |
| 9. Were the exposure measures (independent variables) clearly defined, valid, reliable, and implemented consistently across all study participants? | + |  |  |
| 10. Was the exposure(s) assessed more than once over time? | + |  |  |
| 11. Were the outcome measures (dependent variables) clearly defined, valid, reliable, and implemented consistently across all study participants? | + |  |  |
| 12. Were the outcome assessors blinded to the exposure status of participants? |  |  | NR |
| 13. Was loss to follow-up after baseline 20% or less? | + |  |  |
| 14. Were key potential confounding variables measured and adjusted statistically for their impact on the relationship between exposure(s) and outcome(s)? |  | - |  |

**Bias assessment of case series**

1. Doi 6/9

| **Criteria** | **Yes** | **No** | **Other (CD, NR, NA)*** |
| --- | --- | --- | --- |
| 1. Was the study question or objective clearly stated? |  | - |  |
| 2. Was the study population clearly and fully described, including a case definition? | + |  |  |
| 3. Were the cases consecutive? |  |  | NR |
| 4. Were the subjects comparable? | + |  |  |
| 5. Was the intervention clearly described? | + |  |  |
| 6. Were the outcome measures clearly defined, valid, reliable, and implemented consistently across all study participants? | + |  |  |
| 7. Was the length of follow-up adequate? | + |  |  |
| 8. Were the statistical methods well-described? |  | - |  |
| 9. Were the results well-described? | + |  |  |

1. Irie 6/9

| **Criteria** | **Yes** | **No** | **Other (CD, NR, NA)*** |
| --- | --- | --- | --- |
| 1. Was the study question or objective clearly stated? | + |  |  |
| 2. Was the study population clearly and fully described, including a case definition? | + |  |  |
| 3. Were the cases consecutive? |  |  | NR |
| 4. Were the subjects comparable? | + |  |  |
| 5. Was the intervention clearly described? | + |  |  |
| 6. Were the outcome measures clearly defined, valid, reliable, and implemented consistently across all study participants? | + |  |  |
| 7. Was the length of follow-up adequate? | + |  |  |
| 8. Were the statistical methods well-described? |  | - |  |
| 9. Were the results well-described? |  | - |  |

CD, cannot determine; NA, not applicable; NR, not reported
