# Supplementary material for: Favipiravir versus other antiviral or standard of care for COVID-19 treatment: a rapid systematic review and meta-analysis
Source: Virol J. 2020 Sep 24;17:141. doi: 10.1186/s12985-020-01412-z (PMC7512218; doi:10.1186/s12985-020-01412-z)
Supplement: Supplementary file 3 — Synthesis and sensitivity assessment [file 12985_2020_1412_MOESM3_ESM.docx]

**Synthesis**

**Supplementary file -3**

Subgroup assessment for Virological Clearance using inverse variance method used and showed no significant changes. (Day 7: RR 1.13, 95% CI 0.57 to 2.27; participants = 159; studies = 3; I^2^ = 83%; Day 14: RR 1.06, 95% CI 0.84 to 1.33; participants = 159; studies = 3; I^2^ = 67%)


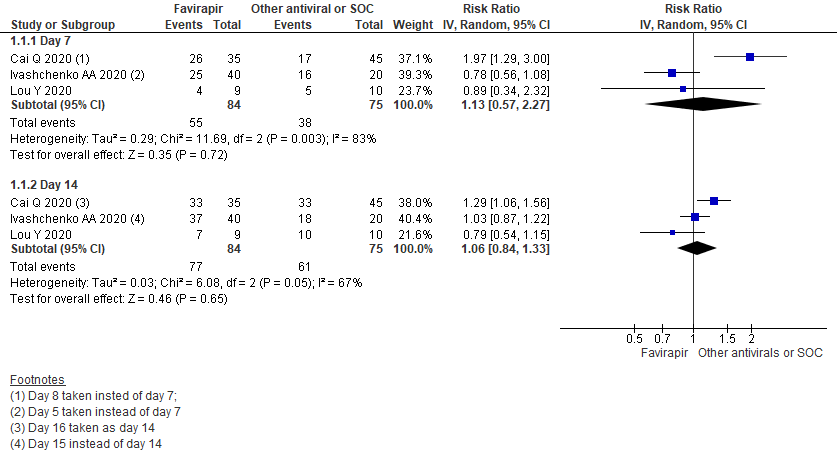


**Figure 1: Forest plot of Sensitivity assessment for Virological Clearance using inverse variance method.**

Sensitivity analysis on virological clearance among two randomized controlled trial excluding non-randomized study by Cai Q et al. [[18](https://www.ncbi.nlm.nih.gov/pmc/articles/PMC7185795/)] (Day 7: RR 0.79, 95% CI 0.58 to 1.08; participants = 79; studies = 3; I^2^ = 0%; Day 14: RR 0.95, 95% CI 0.74 to 1.22; participants = 79; studies = 3; I^2^ = 41%)


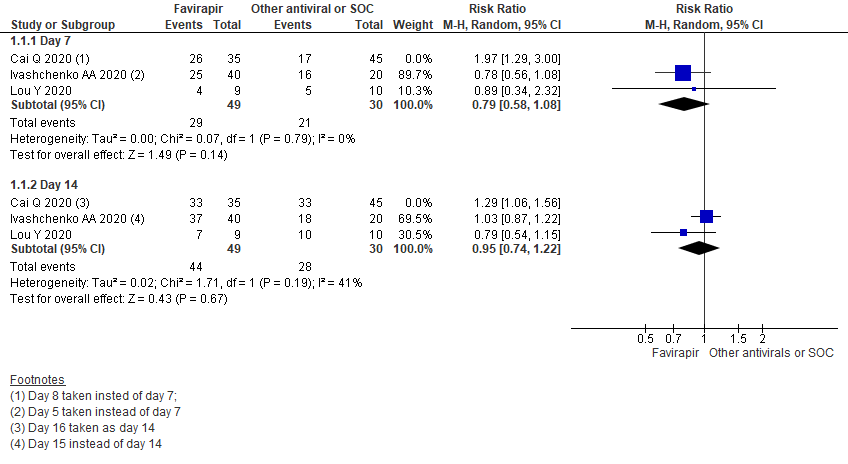


**Figure 2: Forest plot of Sensitivity assessment for Virological Clearance excluding non-randomized study by Cai Q et al** [[18](https://www.ncbi.nlm.nih.gov/pmc/articles/PMC7185795/)]

Clinical improvement on 7^th^ and 14^th^ day among two randomized controlled trial excluding non-randomized study by Cai Q et al. [[18](https://www.ncbi.nlm.nih.gov/pmc/articles/PMC7185795/)] showed slight improvement among favipiravir arm but of no statistical significance (Day 7: RR 1.20, 95% CI 0.96 to 1.50; participants = 255; studies = 3; I^2^ = 0%; Day 14: RR 1.12, 95% CI 0.87 to 1.44; participants = 79; studies = 3; I^2^ = 0%) (Day 7: RD 0.10, 95% CI -0.02 to 0.22; participants = 255; studies = 3; I^2^ = 0%; Day 14: RD 0.09, 95% CI -0.10 to 0.28; participants = 79; studies = 3; I^2^ = 0%).


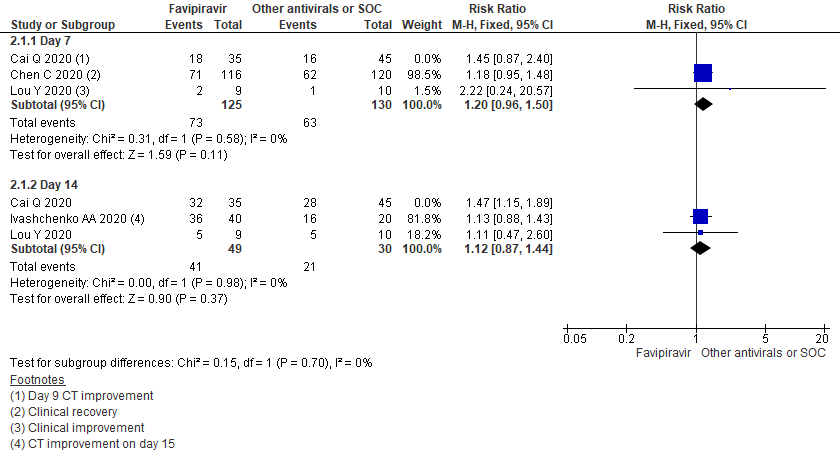

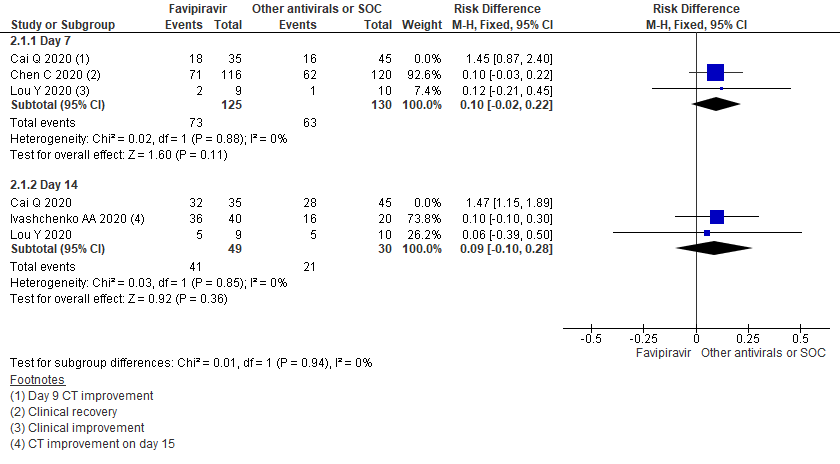


**Figure 3: Forest plot for risk ratios and risk differences regarding FVP in addition to SOC effectiveness for clinical improvement compared with other antivirals or SOC after exclusion of Cai Q et al.** [[18](https://www.ncbi.nlm.nih.gov/pmc/articles/PMC7185795/)]

Overall adverse effects among randomized controlled trials after excluding non-randomized study by Cai Q et al [[18](https://www.ncbi.nlm.nih.gov/pmc/articles/PMC7185795/)] showed slight increase in adverse effects among favipiravir arm but statistically not significant (OR 1.58, 95% CI 0.94 to 2.66; participants = 296; studies = 3; I^2^ = 0%).


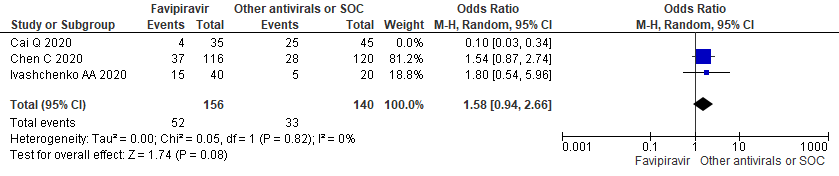


**Figure 4: Forest plot for odds of adverse effects among FVP in addition to versus other antivirals or SOC after exclusion of Cai Q et al.** [[18](https://www.ncbi.nlm.nih.gov/pmc/articles/PMC7185795/)]

Duration to convert Negative RT-PCR: sensitivity assessment using random effect model (MD -2.16, 95% CI -13.28 to 8.97; participants = 99; studies = 2; I^2^ = 45%)


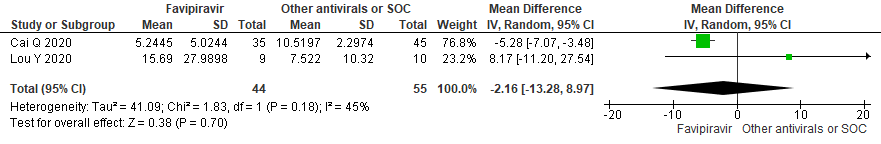


**Figure 5: Forest plot of Favipiravir in addition to standard of care or other anti-virals on Negative conversion of RT-PCR**
