# Supplementary material for: Favipiravir versus other antiviral or standard of care for COVID-19 treatment: a rapid systematic review and meta-analysis
Source: Virol J. 2020 Sep 24;17:141. doi: 10.1186/s12985-020-01412-z (PMC7512218; doi:10.1186/s12985-020-01412-z)
Supplement: Supplementary file 4 — Clinical trials [file 12985_2020_1412_MOESM4_ESM.docx]

**Table**: Current trials on Favipiravir for COVID-19 treatment

| **SN** | **Trial ID** | **Title** | **Enrolled/expected cases** | **Study type** | **Started date/ Completed date** | **Location** | **Primary outcome** |
| --- | --- | --- | --- | --- | --- | --- | --- |
| **Completed** | | | | | | | |
| 1 | NCT04349241 | [Efficacy and Safety of  Favipiravir in Management of  COVID-19](https://clinicaltrials.gov/ct2/show/NCT04349241?term=Favipiravir&recrs=e&cond=COVID-19&draw=2&rank=1) | 100 | CT | 18 Apr 2020 – 20 Jun 2020 | Egypt | Viral clearance (TF:14 d)  Clinical improvement (TF:14 d) |
| 2 | NCT04376814 | Favipiravir [Plus Hydroxychloroquine and Lopinavir/Ritonavir Plus Hydroxychloroquine in COVID-19](https://clinicaltrials.gov/ct2/show/NCT04376814?term=Favipiravir&recrs=e&cond=COVID-19&draw=2&rank=2) | 40 | CT | 29 Mar – 25 May 2020 | Iran | Mortality (TF: up to 28 d)  LoHS (TF: up to 28 d)  Laboratory treatment response: Blood cell count (TF: up to 28 d) & CRP (TF: up to 28 d)  Dyspnea (TF: up to 28 d)  Oxygen saturation without supplemental oxygen. (TF: up to 28 d)  Oxygen therapy (TF: up to 28 d) |
| 3 | NCT04444986 | [Bioequivalence Study of Favipiravir 200 mg Film Tablet Kocak Under Fasting Conditions](https://clinicaltrials.gov/ct2/show/NCT04444986?term=Favipiravir&recrs=e&cond=COVID-19&draw=2&rank=3) | 30 | CT | 05 Jun 2020 -13 Jun 2020 | Turkey | AUC_0-tlast_  and Cmax of Favipiravir (TF: 0 to 24 hours post-dose) |
| 4 | NCT04406194 | [Bioequivalence Study of Favipiravir 200 mg Film Tablet (ATABAY, Turkey) Under Fasting Conditions](https://clinicaltrials.gov/ct2/show/NCT04444986?term=Favipiravir&recrs=e&cond=COVID-19&draw=2&rank=3) | 30 | CT | 14 May – 19 June 2020 | Turkey | AUC_0-tlast_  and Cmax of Favipiravir (TF: 0 to 24 hours post-dose) |
| 5 | NCT04400682 | [Bioequivalence Study of Favipiravir 200 mg Film Tablet (Novelfarma, Turkey) Under Fasting Conditions](https://clinicaltrials.gov/ct2/show/NCT04444986?term=Favipiravir&recrs=e&cond=COVID-19&draw=2&rank=3) | 30 | CT | 28 May – 18 June 2020 | Turkey | AUC_0-tlast_  and Cmax of Favipiravir (TF: 0 to 24 hours post-dose) |
| 6 | NCT04407000 | [Bioequivalence Study of Favipiravir 200 mg Film Tablet (World Medicine, Turkey) Under Fasting Conditions](https://clinicaltrials.gov/ct2/show/NCT04444986?term=Favipiravir&recrs=e&cond=COVID-19&draw=2&rank=3) | 30 | CT | 23 June – 8 August 2020 | Turkey | AUC_0-tlast_  and Cmax of Favipiravi (Time frame 12 and 13 weeks) |

| **SN** | **Trial ID** | **Title** | **Enrolled/expected cases** | **Study type** | **Start date** | **Location** | **Primary outcome** |
| --- | --- | --- | --- | --- | --- | --- | --- |
| **Recruiting** | | | | | | | |
| 1 | NCT04474457 | Efficacy and safety of Favipiravir in the Treatment of COVID-19 Patients Over 15 years of age | 1000 | Prospective observational cohort | 11 June 2020 | Turkey | Time to recovery  Decrease in viral load |
| 2 | NCT04358549 | [Study of the Use of Favipiravir in Hospitalized Subjects With COVID-19](https://clinicaltrials.gov/ct2/show/NCT04358549?term=Favipiravir&recrs=a&cond=COVID-19&draw=2&rank=1) | 50 | CT | 17 Apr 2020 | US | Time to viral clearance (TF: d-29) |
| 3 | NCT04387760 | [Favipiravir vs Hydroxychloroquine in COVID -19](https://clinicaltrials.gov/ct2/show/NCT04387760?term=Favipiravir&recrs=b&cond=COVID-19&draw=2&rank=5) | 150 | CT | 14 Jul 2020 | Bahrain | Primary outcome measure will be time to viral clearance (TF: through study completion up to 21 d) |
| 4 | NCT04392973 | [FAvipiravir and HydroxyChloroquine Combination Therapy](https://clinicaltrials.gov/ct2/show/NCT04392973?term=Favipiravir&recrs=a&cond=COVID-19&draw=2&rank=4) | 520 | CT | 21 May 2020 | Saudi Arabia | Clinical improvement (TF: 28 d)  Viral shedding (TF: 28 d) |
| 5 | NCT04402203 | [Study on Safety and Efficacy of Favipiravir (Favipiravir) for COVID-19 Patient in Selected Hospitals of Bangladesh](https://clinicaltrials.gov/ct2/show/NCT04402203?term=Favipiravir&recrs=a&cond=COVID-19&draw=2&rank=2) | 50 | CT | May 2020 | Bangladesh | Number of participants negative by RT-PCR for the virus at 4-10 d after initiation of therapy. (TF: at 4 to 10 d of therapy)  Number of participants with lung condition change assessed with X-ray. (TF: at d-4, d-7 and d-10 of therapy) |
| 6 | NCT04351295 | [Efficacy of Favipravir in COVID-19 Treatment](https://clinicaltrials.gov/ct2/show/NCT04351295?term=Favipiravir&recrs=b&cond=COVID-19&draw=2&rank=7) | 40 | CT | 17 Apr 2020 | Egypt | Number of patients with viral cure (TF: 6 months) |
| 7 | NCT04411433 | [Efficacy and Safety of Hydroxychloroquine and Favipiravir in the Treatment of Mild to Moderate COVID-19](https://clinicaltrials.gov/ct2/show/NCT04411433?term=Favipiravir&recrs=a&cond=COVID-19&draw=2&rank=3) | 1000 | CT | 08 May 2020 | Turkey | Time to recovery (discharge) (TF: 14 d)  Decrease in viral load (TF: 14 d) |
| 8 | NCT04303299 | [Various Combination of Protease Inhibitors, Oseltamivir, Favipiravir, and Hydroxychloroquine for Treatment of COVID-19 : A Randomized Control Trial](https://clinicaltrials.gov/ct2/show/NCT04303299?term=Favipiravir&recrs=b&cond=COVID-19&draw=2&rank=6) | 320 | CT | 15 Jul 2020 | Thailand | SARS-CoV-2 eradication time (TF: up to 24 weeks) |
| 9 | NCT04445467 | [An Adaptive Randomised Placebo Controlled Phase II Trial of Antivirals for COVID-19 Infection](https://clinicaltrials.gov/ct2/show/NCT04445467?term=Favipiravir&recrs=b&cond=COVID-19&draw=2&rank=8) | 190 | CT | July 2020 | NP | Time to virological cure (TF: 14 d) |
| 10 | NCT04310228 | [Favipiravir Combined With Tocilizumab in the Treatment of Corona Virus Disease 2019](https://clinicaltrials.gov/ct2/show/NCT04310228?term=Favipiravir&recrs=a&cond=COVID-19&draw=2&rank=5) | 150 | CT | 08 Mar 2020 | China | Clinical cure rate (TF: 3 months) |
| 11 | NCT04373733 | [Early Intervention in COVID-19: Favipiravir Verses HydroxycholorquiNe & Azithromycin & Zinc vErsEs Standard CaRe](https://clinicaltrials.gov/ct2/show/NCT04373733?term=Favipiravir&recrs=a&cond=COVID-19&draw=2&rank=7) | 450 | CT | 01 May 2020 | UK | Time to improvement by two points on a seven-category ordinal scale (TF: up to 28 d from randomization) |
| 12 | NCT04333589 | [Corona Virus Disease 2019 Patients Whose Nucleic Acids Changed From Negative to Positive](https://clinicaltrials.gov/ct2/show/NCT04333589?term=Favipiravir&recrs=a&cond=COVID-19&draw=2&rank=6) | 210 | CT | 01 Apr 2020 | China | Viral nucleic acid test negative conversion rate (TF: 5 months) |
| 13 | NCT04403477 | Convalescent Plasma Therapy in Severe COVID-19 Infection | 20 | CT | 20 May 2020 | Bangladesh | Proportion of in-hospital mortality  Time to death |
| 14 | NCT04405310 | Convalescent Plasma of Covid-19 to Treat SARS-COV-2 a Randomized Double Blind 2 Center Trial (CPC-SARS) | 80 | CT | 20 May 2020 | Mexico | Death |

| **SN** | **Trial ID** | **Title** | **Enrolled/expected cases** | **Study type** | **Start date** | **Location** | **Primary outcome** |
| --- | --- | --- | --- | --- | --- | --- | --- |
| **Not Yet Recruiting** | | | | | | | |
| 1 | NCT04464408 | Favipiravir Therapy in Adults with Mild COVID-19 (Avi-Mild) | 578 | CT | July 2020 | Saudi Arabia | PCR Negative |
| 2 | NCT04359615 | [Favipiravir in Hospitalized COVID-19 Patients](https://clinicaltrials.gov/ct2/show/NCT04359615?term=Favipiravir&recrs=b&cond=COVID-19&draw=2&rank=1) | 40 | CT | 20 Apr 2020 | Iran | Time to clinical improvement (TF: from date of randomization until 14 d later) |
| 3 | NCT04425460 | [A Multi-center,Randomized,Double-blind,Placebo-controlled,Phase 3 Study Evaluating Favipiravir in Treatment of COVID19](https://clinicaltrials.gov/ct2/show/NCT04425460?term=Favipiravir&recrs=b&cond=COVID-19&draw=2&rank=2) | 256 | CT | June 2020 | China | Time from randomization to clinical recovery (TF: 28 d) |
| 4 | NCT04475991 | Safety and Efficacy of Maraviroc and/or Favipiravir vs Currently Used Therapy in Severe COVID-19 Adults (COMVIVIR) | 100 | CT | August 2020 | Mexico | Patients free of mechanical ventilation or death |
| 5 | NCT04448119 | [Control of COVID-19 Outbreaks in Long Term Care](https://clinicaltrials.gov/ct2/show/NCT04448119?term=Favipiravir&recrs=b&cond=COVID-19&draw=2&rank=3) | 760 | CT | June 2020 | NP | Control of outbreak (TF: d-40) |
| 6 | NCT04499677 | FLARE: Favipiravir +- Liponavir: A RCT of Early Antivirals (FLARE) | 240 | CT | 05 August, 2020 | London | Upper Respiratory Tract Viral Load at Day 5 |

| **SN** | **Trial ID** | **Title** | **Enrolled/expected cases** | **Study type** | **Start date** | **Location** | **Primary outcome** |
| --- | --- | --- | --- | --- | --- | --- | --- |
| **Active, Not Recruiting** | | | | | | | |
| 1 | NCT04336904 | [Clinical Study To Evaluate The Performance And Safety Of Favipiravir in COVID-19](https://clinicaltrials.gov/ct2/show/NCT04336904?term=Favipiravir&recrs=dfghim&cond=COVID-19&draw=2&rank=1) | 100 | CT | 25 Mar 2020 | Italy | Time from randomization to clinical recovery (TF: 90 d) |
| 2 | NCT04434248 | [An Adaptive Study of Favipiravir Compared to Standard of Care in Hospitalized Patients With COVID-19](https://clinicaltrials.gov/ct2/show/NCT04434248?term=Favipiravir&recrs=dfghim&cond=COVID-19&draw=2&rank=2) | 330 | CT | 23 Apr 2020 | Russian Federation | Rate of viral elimination by d-10 (TF: 10 d)  Time to viral elimination (TF: 28 d)  Time to clinical improvement (TF: 28d) |
| 3 | NCT04501783 | Study of Efficacy and Safety of TL-FVP-t vs. SOC in Patients with Mild to Moderate COVID-19 | 168 | CT | 20 May 2020 | Russia | Time to clinical improvement (through Day28)  Time to viral clearance (through Day 28) |
| 4 | NCT04406194 | An Open Non-comparative Study of the Efficacy and Safety of Aprotinin in Patients Hospitalized With [COVID-19](https://clinicaltrials.gov/ct2/show/NCT04406194?term=Favipiravir&recrs=dfghim&cond=COVID-19&draw=2&rank=5) | 30 | CT | 11 June 2020 | Russia | Time to CRP normalization  Time to d-dimer normalization  Time to elimination of SARS-CoV-2 |

Abbreviations – Apr: April, AUC: Area under curve, Cmax: Maximum plasma concentration, CRP: C-reactive protein, CT: Clinical trial, D: Day, Jul: July, Jun: June, LoHOS: Length of hospital stay, Mar: March, NP: Not provided, PK: Pharmacokinetics, RT-PCR: Reverse transcription polymerase chain reaction, SARC-CoV-2: Severe acute respiratory syndrome coronavirus-2, TF: Time frame, UK: United Kingdom, US: United States.
